# Dr. Percy Boulton

**Published:** 1909-05-22

**Authors:** 


The death has lately occurred, at the age of 69, of Dr. Percy Boulton, M.D., M.R.C.P., the well-known obstetrician and gynæcologist. His father was the late Dr. R. G. Boulton, M.D., J.P., of Beverley, Yorkshire, and he was educated at Beverley Grammar School and Edinburgh University, where he took the M.D. degree in 1862. Sixteen years later he obtained the membership of the London College of Physicians. He practised at first in Nottinghamshire, but came to London in 1865, and was for 30 years physician to the Samaritan Free Hospital for Women and Children. Dr. Boulton was Vice-President of the Obstetrical Society of London, and for a short time was physician to Queen Charlotte's Lying-in Hospital. For eight years he edited the Obstetrical Society's "Transactions," and he was an Examiner and Chairman of the Examining Board of that Society. He was also consulting physician to the British Home for Incurables. Dr. Boulton was an early and ardent worker in the cause of compulsory registration for midwives, and was the author of a number of obstetrical papers and the editor of the fourth edition of "Tanner's Index of Diseases."

